# Effects of Blanc de Noirs and Traditional Red Vinification on the Volatile Composition and Estimated Aroma Potential of Malbec, Bobal, and Tempranillo Wines

**DOI:** 10.3390/foods15213999

**Published:** 2026-11-07

**Authors:** Eva Sánchez-Palomo, María Osorio Alises, Miguel Ángel González Viñas

**Affiliations:** 1 Area of Food Technology, Faculty of Chemical Sciences and Technologies, University of Castilla-La Mancha (UCLM), Avda. Camilo José Cela, 10, 13071 Ciudad Real, Spain; eva.sanchez@uclm.es (E.S.-P.);; 2 Institute for Applied Scientific Research (IRICA), University of Castilla-La Mancha, Avda Camilo José Cela 10, 13071 Ciudad Real, Spain

**Keywords:** Blanc de noirs, Malbec, Bobal, Tempranillo, volatile compounds, odour activity values, wine diversification, warm-climate winemaking

## Abstract

Climate-related changes in grape ripening and composition have increased interest in alternative winemaking approaches in Mediterranean regions. This study evaluated the effects of blanc de noirs and traditional red vinification on the physicochemical characteristics, volatile composition, and estimated aroma potential of wines produced from Malbec, Bobal, and Tempranillo grapes grown in Castilla-La Mancha, Spain. Must and wine physicochemical parameters were determined using standard analytical methods, and volatile compounds were analysed by gas chromatography-mass spectrometry. The effects of grape variety, vinification method, and their interaction on wine composition were assessed by two-way analysis of variance, while odour activity values were calculated to estimate the potential aroma relevance of individual volatile compounds. The physicochemical characteristics of the wines were mainly affected by vinification method. Both grape variety and vinification method significantly affected grape-derived and fermentation-derived volatile compounds, with frequent interactions indicating cultivar-dependent responses. Red vinification generally increased the concentrations of C_6_ compounds, terpenes, C_13_-norisoprenoids, benzenic compounds, higher alcohols, and volatile acids, whereas ester responses differed among cultivars. Total ester concentrations increased in Malbec and Bobal but decreased in Tempranillo after red vinification. Based on their OAVs, β-damascenone and several benzenic compounds were among the compounds with the greatest estimated aroma relevance in Malbec. C_6_ compounds and esters contributed substantially to the OAV-based profiles of Bobal, whereas blanc de noirs Tempranillo wines showed a higher contribution of fruity ethyl esters than red Tempranillo. Blanc de noirs and traditional red vinification produced differentiated volatile profiles, and the magnitude and direction of these changes depended on grape cultivar. These findings provide a compositional basis for future studies evaluating the physicochemical, sensory, technological, and commercial suitability of blanc de noirs wines produced from these cultivars.

## 1. Introduction

Climate change is increasingly recognised as one of the main challenges facing the global wine sector, particularly in Mediterranean wine-growing regions characterised by limited water availability and high thermal stress. In Castilla-La Mancha, the largest wine-producing region in Spain, increasing temperatures, changes in rainfall patterns, and more frequent extreme weather events may accelerate grape ripening and modify grape composition [1,2,3,4]. Grapes grown under warm conditions may reach technological maturity earlier, with increased sugar accumulation, reduced acidity, and changes in phenolic and aroma-precursor composition [5,6]. These modifications can affect wine alcohol content, acidity, volatile composition, and sensory typicity, creating technological challenges for producers in warm-climate regions [7,8].

Different viticultural and oenological practices have been proposed to address these challenges. Viticultural approaches include canopy management, irrigation optimisation, adjustment of harvest timing, and the selection of grape cultivars with greater tolerance to heat and water stress. Oenological approaches include modifications to fermentation and processing conditions and the development of alternative wine styles [9]. Among these alternatives, the blanc de noirs vinification of red grape cultivars can produce wines with limited colour extraction and volatile profiles that differ from those obtained through traditional red vinification [10,11]. However, its potential contribution to climate adaptation or regional wine diversification requires evaluation beyond compositional analysis, including physicochemical, sensory, technological, and economic assessment.

Blanc de noirs vinification generally involves pressing the grapes shortly after harvest and minimising contact between the juice and grape skins. Limited skin contact restricts the extraction of anthocyanins, tannins, and other phenolic compounds, resulting in wines with lower colour intensity and phenolic content than traditionally produced red wines [12,13]. This vinification method may also modify the extraction, formation, retention, and loss of volatile compounds and their precursors. Consequently, the volatile composition of blanc de noirs wines may differ from that of traditional red wines produced from the same grape material [10,14]. Nevertheless, compositional differences alone cannot establish sensory freshness, balance, quality, consumer acceptance, or suitability for a particular wine style.

Castilla-La Mancha provides an appropriate context for investigating the response of red grape cultivars to alternative vinification methods because red cultivars account for a substantial proportion of its vineyard area and are exposed to warm and dry growing conditions. The present study focused on Tempranillo, Malbec, and Bobal, three cultivars with contrasting agronomic and oenological characteristics. Tempranillo is one of the most widely cultivated Spanish grape varieties and is characterised by relatively early ripening, which may increase its susceptibility to accelerated sugar accumulation under high-temperature conditions [15]. Malbec, although traditionally associated with other wine-producing regions, can develop a distinctive volatile composition when cultivated in Castilla-La Mancha [16]. Bobal is an autochthonous cultivar from eastern and central Spain that is recognised for its adaptation to dry conditions and its high phenolic potential [17]. These varietal characteristics may influence the extraction and formation of volatile compounds under blanc de noirs and red vinification conditions.

Comparing these cultivars under the same experimental vinification conditions makes it possible to determine whether the effect of the vinification method depends on grape variety. Such a comparison can provide information about the compositional responses of each cultivar. However, it does not demonstrate the sensory suitability, commercial potential, or climate-adaptation capacity of the resulting wines. These aspects require complementary studies incorporating physicochemical characterisation, sensory analysis, consumer evaluation, different harvest maturity stages, and multiple vintages.

Wine aroma is an important component of wine identity and consumer perception and is determined by a complex mixture of grape-derived and fermentation-derived volatile compounds [18,19]. In blanc de noirs wines, reduced skin contact and differences in oxidation, precursor extraction, and fermentation dynamics may produce volatile profiles that differ substantially from those of traditional red wines [10]. Previous studies have demonstrated that grape variety and winemaking conditions influence the concentrations of higher alcohols, esters, volatile fatty acids, terpenes, C_13_-norisoprenoids, benzenic compounds, and other volatile compounds [11,20].

Odour activity values can be used to estimate the potential aroma relevance of individual compounds by relating their concentrations to their reported odour thresholds. However, OAVs do not constitute a direct sensory measurement and cannot fully account for interactions among volatile compounds or for the influence of the wine matrix. Therefore, OAV-based results should be interpreted as estimates of potential aroma contribution rather than as evidence of perceived aroma intensity, sensory balance, quality, or consumer acceptance.

Accordingly, this study aimed to evaluate the effects of blanc de noirs and traditional red vinification on the volatile composition and estimated aroma potential of wines produced from Tempranillo, Malbec, and Bobal grapes grown in Castilla-La Mancha. The effects of grape variety, vinification method, and their interaction were examined to determine whether the response to vinification was cultivar-dependent. We hypothesised that blanc de noirs and red vinification would produce different volatile profiles and that the magnitude and direction of these differences would depend on grape cultivar.

## 2. Materials and Methods

### 2.1. Winemaking Process

Grapes were manually harvested in 2025 according to the technological maturity of each cultivar. Malbec and Bobal were harvested on 17 August, whereas Tempranillo was harvested on 25 August. All cultivars were grown in the same rainfed, bush-trained vineyard located in the municipality of Manzanares, Castilla-La Mancha, Spain. For each cultivar, the entire batch of harvested grapes was thoroughly mixed before processing to minimise heterogeneity in the initial grape material. The homogenised grapes were subsequently divided into equivalent lots, which were assigned to blanc de noirs or traditional red vinification. Thus, within each cultivar, both vinification methods were applied to grapes from the same vineyard plot, harvest date, and initial maturity stage. Two independent vinifications were carried out for each combination of cultivar and vinification method.

#### 2.1.1. Blanc de Noirs Vinification

Approximately 25 kg of fruit per sample were collected in 10 kg crates and immediately transported to the laboratory. The grapes underwent destemming and crushing, followed by gentle pressing in a pneumatic press to extract the juice with minimal skin contact. The resulting must was sulphited (100 mg/L SO_2_) and transferred to 50 L vessels for static settling at 10 ± 2 °C for 24 h. After the removal of residual solids, the clear juice was moved to fermentation tanks. Alcoholic fermentation was conducted at a controlled temperature of 18 ± 2 °C, using *Saccharomyces cerevisiae* (UCLM S325, Fould-Springer) as the inoculant. Once fermentation was complete (roughly 1 week), the wines were racked, clarified through 0.45 µm membrane filters (Millipore), bottled, and stored in a temperature-controlled environment at 8–10 °C. All experimental wines were produced and analysed during the same harvest year.

#### 2.1.2. Traditional Red Vinification

After destemming and crushing, laboratory-scale fermentations were performed in duplicate using 10 L glass vessels, following the standard red winemaking protocol with skin contact. Following the removal of stems and crushing, the must was treated with 100 mg/L of SO_2_. Alcoholic fermentation was initiated by inoculating the selected *Saccharomyces cerevisiae* yeast strain (UCLM S325, Fould-Springer) at a steady temperature of 24 °C. During this phase, the cap was manually punched down every 12 h. The wine was separated from the solid parts once the relative density stabilised at 0.995 g/L. Malolactic fermentation was subsequently induced using *Oenococcus oeni* (Lactobacter SP1; Laffort) and tracked through thin-layer chromatography (TLC) until completion, which typically took 2–3 weeks. Afterward, wines were racked, passed through 1.2 µm membrane filters (Millipore), bottled, and stored in a temperature-controlled cellar at 16–18 °C.

### 2.2. Physicochemical Characteristics of Musts and Wines

The physicochemical characteristics of the musts and wines were determined according to the methods established by the International Organisation of Vine and Wine (OIV) in the Compendium of International Methods of Wine and Must Analysis [21]. Soluble solids (°Brix) and pH were determined in the musts before fermentation. After vinification, the wines were analysed for pH, titratable acidity (TA), ethanol content, volatile acidity (VA), and free and total SO_2_ concentrations.

### 2.3. Volatile Compounds

The aromatic profile of the wines was determined by analysing volatile compounds. Wine samples (100 mL) were passed through 0.5 g Supelclean Envi-Chrom P cartridges (Sigma-Aldrich, St. Louis, MO, USA) at a flow rate of 1 mL min^−1^. To remove residual sugars and other polar low-molecular-weight constituents, the cartridges were rinsed with 50 mL of Milli-Q water. The retained volatile fraction was subsequently eluted using 10 mL of dichloromethane, and the resulting extract was concentrated under a gentle stream of nitrogen to a final volume of 200 μL.

Chromatographic analyses were carried out using an Agilent 6890N gas chromatograph coupled to an Agilent 5973 inert mass selective detector (Agilent Technologies, Santa Clara CA, USA). Separation was achieved on a DB-WAX (60 m × 0.25 mm × 0.25 μm) (Agilent Technologies, Santa Clara, CA, USA). A volume of 1 μL of extract was injected, while helium was employed as carrier gas at a constant flow rate of 1 mL min^−1^. The oven temperature programme started at 70 °C and was maintained for 5 min, followed by an increase at 1 °C min^−1^ to 95 °C with a subsequent hold of 10 min. The temperature was then raised at 2 °C min^−1^ to 200 °C and maintained for 40 min. The injector temperature was set at 250 °C. Mass spectrometric detection was performed in electron impact (EI) mode at 70 eV. Data acquisition was conducted in full-scan mode over a mass range of 40–450 m/z, with the ion source temperature maintained at 280 °C.

Volatile compounds were identified by comparing their retention times and mass spectra with those of authentic standards. When authentic standards were unavailable, tentative identification was based on comparison of the acquired mass spectra with those included in the Wiley G1035A and NBS75K mass spectral libraries. Quantification was performed using 4-nonanol as the internal standard. Compounds for which authentic standards were available were quantified using compound-specific calibration curves based on the analyte-to-internal-standard response ratio. Compounds lacking authentic standards were semi-quantified relative to 4-nonanol by assuming a response factor of 1, and their concentrations should therefore be considered estimates. Calibration parameters, the standard used, the quantification approach, and identification information are provided in Supplementary Table S1 for varietal volatile compounds and in Supplementary Table S2 for volatile compounds formed principally during fermentation.

### 2.4. Odour Activity Values (OAVs)

Odour activity values were calculated as the ratio between the concentration of each volatile compound and its corresponding odour threshold reported in the literature [18,22,23]. Compounds with OAVs above 1 were considered potentially active odorants. Compounds with OAVs above 0.1 in at least one wine were included in Table 2 because subthreshold compounds may contribute through additive, synergistic, antagonistic, or matrix-dependent interactions.

### 2.5. Statistical Analysis

Statistical analyses were performed using IBM SPSS Statistics software (version 29.0; IBM Corp., Armonk, NY, USA). Physicochemical parameters of the musts were analysed by one-way analysis of variance (ANOVA), with grape variety (Tempranillo, Malbec, and Bobal) as the fixed factor. When a significant effect was detected, mean values were compared using the Student–Newman–Keuls (SNK) test at *p* < 0.05. For the wines, the effects of grape variety, vinification method (blanc de noirs and red vinification), and their interaction were evaluated by two-way ANOVA. This factorial analysis was applied to the physicochemical parameters and individual volatile compounds of the wines. Effect sizes were expressed as partial eta squared (ηp^2^). Statistical significance was established at *p* < 0.05. Exact *p*-values and ηp^2^ values for the main effects and their interaction are reported in the corresponding tables, with *p*-values below 0.001 expressed as *p* < 0.001.

## 3. Results and Discussion

### 3.1. Physicochemical Characteristics of Musts and Wines

The physicochemical characteristics of the musts and wines are presented in Table 1. Must pH differed significantly among cultivars, with Malbec showing the lowest value and Bobal the highest. In contrast, no significant differences were found in soluble solids or titratable acidity. Overall, these results suggest a broadly comparable technological maturity at harvest in terms of soluble solids and titratable acidity, despite the cultivar-dependent differences observed in must pH. In the wines, the effects of grape variety and vinification method depended on the physicochemical parameter evaluated. Grape variety significantly affected pH, titratable acidity, volatile acidity, and free SO_2_ concentration, whereas the vinification method significantly affected ethanol content, titratable acidity, and total SO_2_ concentration. A significant grape variety × vinification interaction was observed for pH, indicating that the effect of vinification method on this parameter differed among cultivars. No significant interactions were observed for the remaining physicochemical parameters. The partial eta-squared values indicated large effects for the statistically significant factors and for the interaction observed for pH. Overall, these results provide the physicochemical context required to interpret the cultivar-dependent differences in volatile composition associated with blanc de noirs and traditional red vinification.

### 3.2. Effect of Grape Variety and Vinification Method on Varietal Volatile Composition

**Figure 1:**
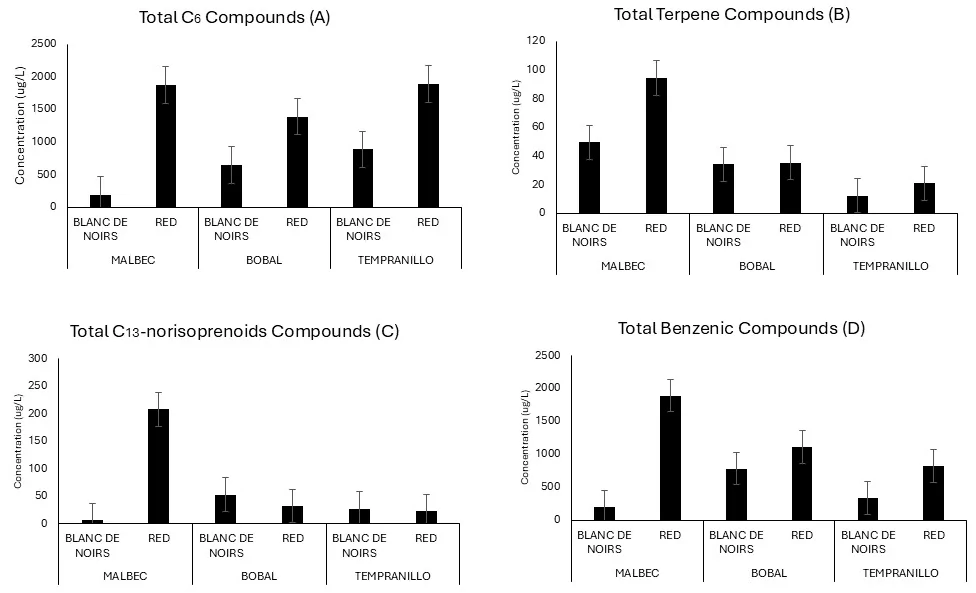
Total concentrations (μg L^−1^) of the main families of grape-derived volatile compounds in wines produced from Malbec, Bobal, and Tempranillo grapes by white and traditional red vinification: (**A**) total C_6_ compounds; (**B**) total terpene compounds; (**C**) total C_13_-norisoprenoids; and (**D**) total benzenic compounds.

Gas chromatography–mass spectrometry (GC-MS) analysis enabled the detection and quantification of 33 grape-derived volatile compounds in blanc de noirs vinification and red wines produced from Tempranillo, Malbec, and Bobal grapes. These compounds were grouped into four major chemical families: C_6_ compounds, terpenes, C_13_-norisoprenoids, and benzenic compounds. The total concentrations of these families are presented in Figure 1A–D, whereas the concentrations of individual compounds and the results of the statistical analysis are reported in Supplementary Table S3. The effects of grape variety, vinification type, and their interaction were evaluated by two-way analysis of variance. This factorial approach allowed the influence of the intrinsic aromatic potential of each cultivar to be distinguished from that of the winemaking procedure. The differences observed between blanc de noirs and red wines reflect the overall effect of the corresponding winemaking protocols, including skin contact, fermentation temperature, malolactic fermentation, filtration, and storage conditions.

Overall, varietal volatile composition was influenced by both grape variety and vinification method. Red wines generally contained higher concentrations of grape-derived compounds because prolonged skin contact favours the extraction of free volatiles and non-volatile precursors, whereas direct pressing during blanc de noirs vinification limits their transfer to the must [24,25]. Glycosylated precursors may subsequently release volatile aglycones through enzymatic or acid-catalysed hydrolysis during fermentation and storage [26]. However, the significant grape variety × vinification interactions observed for several compounds indicate that the effect of vinification depended on the biochemical characteristics of each cultivar. Thus, vinification method mainly modulated the quantitative expression of varietal aroma, while grape variety determined its qualitative profile.

#### 3.2.1. C_6_ Compounds

C_6_ alcohols and aldehydes are abundant grape and must volatiles formed through the lipoxygenase pathway from linoleic and linolenic acids after tissue disruption. Compounds such as hexanal, (E)-2-hexenal, 1-hexanol, (E)-2-hexen-1-ol, and (Z)-3-hexen-1-ol contribute green, herbaceous, cut-grass, and fresh-fruit nuances. Their concentrations depend on cultivar, berry maturity, oxygen availability, and grape-processing conditions, and they are particularly relevant in neutral grape varieties [27].

As shown in Figure 1A, total C_6_ concentrations depended on grape variety and vinification method and were generally higher in red wines, probably because of greater tissue disruption and prolonged skin contact. The importance of C_6_ compounds in Castilla-La Mancha wines agrees with previous studies identifying C_6_ and benzenic compounds as major grape-derived volatile families in Malbec wines and as contributors to the green and fresh character of young red wines from this region [16,28].

Bobal showed a particularly important C_6_ contribution under both vinification conditions, consistent with the predominance of C_6_ alcohols and aldehydes previously reported in Bobal and Tempranillo musts [29]. Although blanc de noirs vinification reduced their total concentration, blanc de noirs Bobal wines retained a substantial C_6_ fraction, indicating that a substantial fraction of its C_6_ volatile composition was retained under limited skin-contact conditions. Tempranillo also showed a relevant C_6_ contribution, although its profile was more evenly distributed between C_6_ and benzenic compounds. Differences previously reported in 1-hexanol and other volatiles between Tempranillo and Tempranillo Blanco further demonstrate the combined influence of grape genotype and winemaking conditions [30].

The ANOVA results reported in Supplementary Table S3 confirm the effects of variety, vinification method, and their interaction. Consequently, the concentrations of varietal volatile compounds associated with green and fresh odour descriptors depend on both skin contact and cultivar-specific precursor composition, although their actual sensory relevance requires sensory confirmation.

**Terpenes**

Terpenes are grape-derived aroma compounds with relatively low odour thresholds that contribute floral, citrus, sweet, and balsamic nuances. Compounds such as linalool, geraniol, nerol, citronellol, and α-terpineol may occur in grapes in free form or as non-volatile glycosides, which can release odoriferous aglycones during fermentation and storage. The composition and concentration of these precursors vary among cultivars, making terpenes important markers of varietal typicity, particularly in aromatic grape varieties [24,26,31].

Terpenes represented a relatively minor fraction of the varietal volatile composition of the wines (Figure 1B), as expected for Tempranillo, Malbec, and Bobal, which are not considered highly aromatic cultivars. Nevertheless, the differences observed among varieties and vinification methods indicate that these compounds may contribute to wine differentiation even at moderate concentrations.

Red wines generally contained higher terpene concentrations than blanc de noirs wines. This trend can be attributed to the preferential location of free and glycosidically bound terpenes in grape skins, whose extraction is enhanced during maceration [25]. Tempranillo showed a limited terpene contribution under both winemaking conditions, consistent with its relatively neutral aromatic character, whereas Malbec and Bobal exhibited distinct but moderate terpene profiles.

The lower terpene content of blanc de noirs wines was probably related to the early separation of the juice from the skins. However, limited skin contact may also reduce oxidative losses, meaning that the final terpene concentration reflects the balance between restricted extraction and improved preservation.

The statistical results reported in Supplementary Table S3 confirm that terpene composition depended on the individual compound, grape variety, and vinification method. Significant interactions between both factors indicate cultivar-specific differences in terpene extraction or retention. Given their low odour thresholds, the sensory relevance of individual terpenes should be evaluated using OAVs rather than total family concentrations alone.

**C_13_-norisoprenoids**

C_13_-norisoprenoids originate from carotenoid degradation and include highly aroma-active compounds such as β-damascenone, β-ionone, and vitispirane. Owing to their low odour thresholds, they can contribute floral, violet, ripe-fruit, dried-fruit, honey, and balsamic notes even at trace concentrations and may also enhance fruity perception through interactions with other odorants. Their expression depends on cultivar, berry development, environmental conditions, and precursor transformations during winemaking [24,31].

Total C_13_-norisoprenoid concentrations differed according to grape variety and vinification method (Figure 1C). Red vinification generally promoted their extraction, which is consistent with the location of carotenoids and related precursors in grape skins and their greater extraction during maceration.

Malbec showed the highest total concentration of this volatile family, particularly after red vinification. This result is consistent with the aromatic complexity and ripe-fruit, floral, sweet, liquorice, and balsamic characteristics previously described for Malbec wines from Castilla-La Mancha [16]. Although blanc de noirs Malbec wines showed a low total concentration of C_13_-norisoprenoids, β-damascenone exhibited a high OAV because of its low odour threshold. Therefore, the potential aroma relevance of this compound cannot be inferred solely from the total concentration of the volatile family. Its presence in blanc de noirs Malbec wines also indicates that some C_13_-norisoprenoid precursors or their volatile degradation products were retained under limited skin-contact conditions.

Tempranillo exhibited a lower C_13_-norisoprenoid concentration, especially after blanc de noirs vinification, whereas Bobal showed an intermediate and compound-dependent response. The ANOVA results reported in Supplementary Table S3 confirmed significant effects of grape variety, vinification method, and their interaction, reflecting cultivar-specific differences in precursor composition and extractability.

Total family concentrations should, however, be interpreted cautiously because individual C_13_-norisoprenoids differ considerably in their odour thresholds and potential aroma relevance. β-Damascenone, for example, may have a potentially relevant contribution even at very low concentrations. Therefore, the high OAV of β-damascenone in blanc de noirs Malbec indicates potentially relevant aroma activity despite the low total concentration of C_13_-norisoprenoids in this wine (Table 2).

#### 3.2.2. Benzenic Compounds

Benzenic compounds constituted a quantitatively important family of grape-derived volatiles (Figure 1D), including benzyl alcohol, benzaldehyde derivatives, and other aromatic ring compounds. However, some may have mixed origins because they can occur naturally in grapes, be released from grape precursors, or be modified by yeast metabolism; their classification should therefore consider their predominant origin within the analytical framework of the study.

Red wines generally contained higher concentrations of benzenic compounds than blanc de noirs wines, consistent with the enhanced extraction of free volatiles and glycosidically bound precursors during maceration. This effect was especially pronounced in Malbec, indicating both a high intrinsic benzenic potential and a strong response to skin contact.

Malbec showed the greatest contribution from this family, in agreement with previous studies identifying C_6_ and benzenic compounds as major groups of free varietal volatiles in Malbec wines from Castilla-La Mancha [16]. These compounds may contribute floral, sweet, balsamic, spicy, and ripe-fruit nuances, although their sensory relevance depends on their individual concentrations and odour thresholds. Together with C_13_-norisoprenoids, they may contribute to the complex sensory profile previously reported for these wines, including red-fruit, prune, liquorice, clove, caramel, leather, tobacco, and coffee notes [16,28].

Blanc de noirs Malbec wines also retained a substantial benzenic fraction, suggesting the presence of abundant or readily extractable precursors and indicating that blanc de noirs vinification retained a substantial fraction of the benzenic volatile composition of Malbec. Tempranillo showed a moderate contribution, whose relative importance was enhanced by its limited terpene content, whereas Bobal showed a stronger contribution from C_6_ compounds associated in the literature with green and herbaceous odour descriptors [29].

The ANOVA results reported in Supplementary Table S3 confirm that benzenic composition depended on grape variety, vinification method, and, for several compounds, their interaction. This was particularly relevant for Malbec, whose benzenic fraction increased markedly after red vinification while remaining comparatively well represented under blanc de noirs vinification.

### 3.3. Effect of Grape Variety and Vinification Method on Volatile Compounds Formed Principally During Alcoholic Fermentation

**Figure 2:**
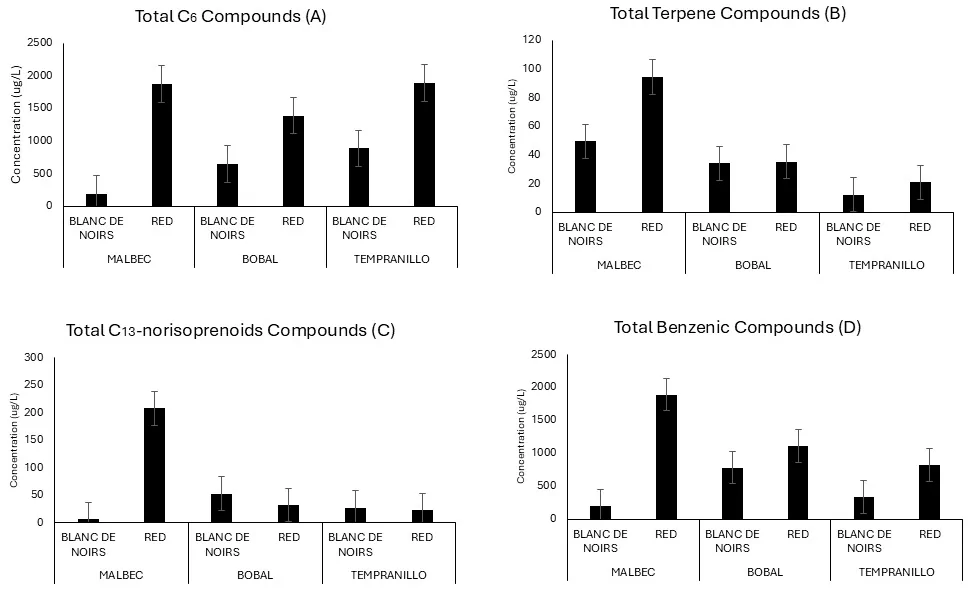
Total concentrations (μg L^−1^) of the main families of volatile compounds predominantly formed during fermentation in wines produced from Malbec, Bobal, and Tempranillo grapes by white and traditional red vinification: (**A**) total volatile acids; (**B**) total higher alcohols; and (**C**) total esters.

The total concentrations of volatile acids, higher alcohols, and esters are shown in Figure 2A, Figure 2B, and Figure 2C, respectively. The values used to construct these graphical representations correspond to the sum of the compounds quantified within each chemical family and the concentrations of the individual volatile compounds and the results of the two-way analysis of variance are presented in Supplementary Table S4. All the individual compounds were significantly affected by grape variety and vinification method, although the magnitude of these effects varied among chemical families. The significant variety × vinification interactions observed for most compounds indicate that the effect of blanc de noirs or red winemaking was cultivar-dependent. Therefore, the differences in volatile composition cannot be attributed exclusively to the greater extraction associated with red vinification. They also reflect differences in must composition, nutrient availability, fermentation metabolism, and, potentially, the occurrence of malolactic fermentation.

This combined effect is consistent with the biochemical origin of these volatile compounds. During fermentation, wine microorganisms transform grape-derived substrates and synthesise a broad range of secondary metabolites, including higher alcohols, esters, and volatile fatty acids. Their concentrations depend on the grape matrix, yeast metabolism, fermentation conditions, and microbial interactions [32,33].

#### 3.3.1. Esters

Although quantitatively less abundant than higher alcohols, esters were particularly relevant because of their low odour thresholds and potential contribution to fruity and floral aroma characteristics (Figure 2C). Red vinification increased total ester concentrations in Malbec and Bobal wines, whereas they decreased in Tempranillo wines. This contrasting response, together with the significant grape variety × vinification interactions observed for several individual esters (Supplementary Table S4), indicates that the effect of vinification method on ester composition was strongly cultivar-dependent.

Acetate esters are mainly formed through the enzymatic condensation of higher alcohols with acetyl-CoA, whereas ethyl esters derive predominantly from reactions between ethanol and fatty-acid-derived acyl-CoA. Their final concentrations depend on precursor availability, yeast strain, nitrogen status, temperature, oxygen exposure, alcohol acyltransferase and esterase activities, and retention within the wine matrix [32,33,34,35].

The ethyl esters ethyl butanoate, ethyl hexanoate, and ethyl octanoate generally increased after red vinification in Malbec and Bobal but decreased in Tempranillo. The simultaneous accumulation of some fatty acids in red Tempranillo indicates that precursor availability alone did not determine ester formation. Instead, the balance among fatty-acid metabolism, acyl-CoA formation, esterification, hydrolysis, and physicochemical retention controlled their final concentrations. In *Saccharomyces cerevisiae*, the enzymes encoded by EEB1 and EHT1 participate in medium-chain fatty acid ethyl ester production, whereas esterases may reduce their accumulation [34,36,37].

Isoamyl acetate was particularly abundant in Bobal and increased after red vinification in Malbec, whereas it showed little variation in Tempranillo. This ester contributes banana, pear drop, and fruity notes, and its formation is regulated mainly by alcohol acetyltransferases, including those encoded by ATF1 and ATF2 [36,38,39]. Conversely, hexyl acetate was consistently more abundant in blanc de noirs wines, confirming that individual esters may respond differently from the total ester family because ester-producing and ester-degrading enzymes differ in substrate specificity [36,37].

The higher concentrations of ethyl lactate and diethyl succinate in some red wines may also be related to the malolactic fermentation performed after alcoholic fermentation [40,41]. Differences in these esters between Tempranillo-derived wines have previously been related to both grape material and malolactic fermentation [30].

The concentration of 2-phenylethyl acetate remained relatively stable in Malbec and Bobal but decreased in traditional Tempranillo. This floral, rose-like, and honeyed ester is produced through the acetylation of 2-phenylethanol. Its decrease despite the accumulation of 2-phenylethanol confirms that precursor abundance alone does not determine ester formation and suggests differences in acetyltransferase activity, acetyl-CoA availability, hydrolysis, or yeast physiology [34,36,38].

Overall, red Bobal exhibited the highest total ester content, whereas blanc de noirs Tempranillo wines showed the greatest ester concentration among the blanc de noirs wines. These differences may produce distinct fruity and floral profiles, although their sensory relevance should be assessed using odour activity values and considering possible additive, synergistic, or suppressive interactions among odorants [32,42]. Thus, the ester fraction confirms that the influence of vinification was cultivar-specific and resulted from the combined effects of grape composition, microbial metabolism, and winemaking conditions.

#### 3.3.2. Acids

Red vinification generally increased the total concentration of volatile acids, although the magnitude of this effect was cultivar-dependent and particularly pronounced in Tempranillo (Figure 2A). The significant grape variety × vinification interactions observed for most compounds confirm that the acid profile was determined by the combined influence of the grape matrix and the winemaking procedure.

Hexanoic, octanoic, and decanoic acids were the predominant compounds. These medium-chain fatty acids arise from yeast lipid metabolism and act as precursors of their corresponding ethyl esters. At moderate concentrations, they may enhance aroma complexity, whereas excessive levels can impart fatty, cheesy, rancid, or soapy nuances. Their sensory contribution depends on their concentrations, odour thresholds, and interactions with esters and other wine constituents [32,33].

Octanoic acid predominated in most wines and increased after red vinification in Malbec and Tempranillo but decreased in Bobal. Hexanoic acid was less affected, whereas decanoic acid showed marked variability, increasing after red vinification in all three cultivars, particularly in Malbec and Tempranillo. Its accumulation contributed substantially to the high total acid content of traditional Tempranillo.

The branched-chain acids isobutyric, isovaleric, and 2-methylbutanoic acid also showed cultivar-dependent responses. These compounds originate mainly from yeast metabolism of branched-chain amino acids and may contribute fatty, cheesy, rancid, or fermented notes above their sensory thresholds. Their formation may reflect differences in amino acid availability and yeast nitrogen metabolism among musts [33,43].

Red wines also generally showed higher levels of (E)-2-hexenoic acid, whereas (E)-3-hexenoic acid exhibited less consistent variations. Overall, red vinification enhanced volatile acid formation, particularly in Tempranillo, although individual responses depended strongly on grape variety. These results agree with previous studies showing that the volatile acid profile of Tempranillo-derived wines reflects the interaction between grape characteristics, winemaking conditions, and microbial metabolism [30].

#### 3.3.3. Alcohols

Higher alcohols were the predominant fermentation-derived volatile family (Figure 2B). Red vinification enhanced their total concentration in Malbec and especially Tempranillo, whereas Bobal maintained similar levels under both winemaking conditions. The significant grape variety × vinification interactions reported in Supplementary Table S4 confirm that their formation depended on the combined effects of grape composition and fermentation conditions.

The differences among wines were mainly attributable to 2-phenylethanol, which was the dominant alcohol. This compound is produced by yeast from phenylalanine through the Ehrlich pathway or by de novo amino acid biosynthesis and contributes floral, rose-like, honeyed, and perfumed notes at appropriate concentrations. Its formation is influenced by yeast strain, nitrogen availability, oxygen exposure, and fermentation conditions [32,43,44,45].

Red vinification promoted 2-phenylethanol formation in Malbec and particularly in Tempranillo, whereas Bobal retained similarly high concentrations under both winemaking conditions. Because higher alcohols are mainly produced by yeast, this response should not be interpreted as direct extraction from grape skins. Instead, skin contact may modify nitrogen and amino acid availability, turbidity, lipid composition, oxygen exposure, and microbial growth, thereby altering yeast metabolism. These relationships are complex and cultivar-dependent [33,45,46]. Differences previously reported between Tempranillo traditional red wines and Blanc de noirs Tempranillo wines further support the combined influence of grape genotype and vinification on higher-alcohol composition [30].

The branched-chain alcohols 2-methyl-1-butanol and 3-methyl-1-butanol generally increased after red vinification, although the magnitude of the response varied among cultivars. These compounds arise from branched-chain amino acids through the Ehrlich pathway or from intermediates of central carbon metabolism, and their formation depends on yeast strain, nitrogen status, oxygen availability, temperature, and precursor concentration [44,45].

The sulphur-containing alcohol 3-methylthio-1-propanol was also enhanced by red vinification. It originates from yeast metabolism of methionine and may contribute cooked-potato, cauliflower, vegetable, or sulphurous notes, depending on its concentration and the wine matrix [43,47,48]. The *levo* and *meso* forms of 2,3-butanediol also varied according to variety and vinification method. Although their direct sensory contribution is limited, their concentrations and stereoisomeric distributions provide information about yeast metabolism and fermentation development [49,50].

Overall, red vinification favoured higher-alcohol formation in Malbec and particularly Tempranillo, whereas Bobal maintained high concentrations under both winemaking conditions. More broadly, variety and vinification jointly shaped both varietal and fermentation-derived volatiles: red Tempranillo was characterised by high alcohol and acid concentrations, red Bobal by its elevated ester content, and Malbec by a consistent increase in most volatile families. The significant interactions between both factors confirm that each cultivar responds differently to vinification, producing distinct aromatic profiles whose sensory relevance should be confirmed through odour activity values and sensory analysis.

### 3.4. Odour Activity Values and Potential Contribution of Volatile Compounds to Wine Aroma

The potential sensory relevance of the volatile compounds was assessed from their odour activity values (OAVs), with values above 1 indicating compounds likely to contribute individually to wine aroma, although the final perception may be modified by interactions among odorants and by the wine matrix [18,23,51]. Table 2 presents the OAVs of the volatile compounds in the blanc de noirs and traditional wines. Among the blanc de noirs wines, Malbec and Bobal were primarily distinguished by the high OAVs of β-damascenone and ethyl octanoate, whereas Tempranillo was dominated by ethyl octanoate, ethyl hexanoate, and ethyl butanoate, with no detectable contribution from β-damascenone. Blanc de noirs Malbec wines showed the highest β-damascenone OAV, blanc de noirs Bobal wines the highest values for isoamyl acetate and 2-phenylethanol, and blanc de noirs Tempranillo wines the highest OAVs for ethyl butanoate and ethyl hexanoate. After red vinification, varietal differentiation became more pronounced: Malbec was clearly dominated by β-damascenone, followed by ethyl octanoate and ethyl hexanoate; Bobal was mainly characterised by ethyl octanoate, isovaleric acid, isoamyl acetate, and ethyl hexanoate; and Tempranillo was mainly characterised by β-damascenone and fruity ethyl esters, together with a relevant but comparatively lower contribution from isovaleric acid, 2-phenylethanol, and guaiacol. Within each variety: Red vinification increased the number and relative importance of potentially active odorants in Malbec, particularly β-damascenone, ethyl hexanoate, isoamyl acetate, 2-phenylethanol, guaiacol, and linalool. In Bobal, red vinification increased ethyl octanoate, ethyl hexanoate, isovaleric acid, and guaiacol but reduced β-damascenone and isoamyl acetate, thereby substantially modifying the odorant hierarchy. Tempranillo showed the greatest reordering, shifting from a blanc de noirs wine profile dominated by fruity ethyl esters to a red wine profile in which β-damascenone, isovaleric acid, butyric acid, 2-phenylethanol, and guaiacol acquired greater potential relevance. Overall, β-damascenone and ethyl octanoate were the most prominent odorants across the wines, while ethyl hexanoate, ethyl butanoate, isoamyl acetate, and several volatile fatty acids formed a second group of potentially relevant compounds, in agreement with previous studies identifying these molecules among the most powerful wine odorants [18,23]. Thus, vinification modified not only the magnitude but also the ranking of odour-active compounds in a cultivar-dependent manner; nevertheless, these OAV-based rankings should be confirmed through sensory analysis, since they do not fully account for enhancement, suppression, or other perceptual interactions among wine odorants [51].

### 3.5. Aromatic Series Calculated from Odour Activity Values

**Figure 3:**
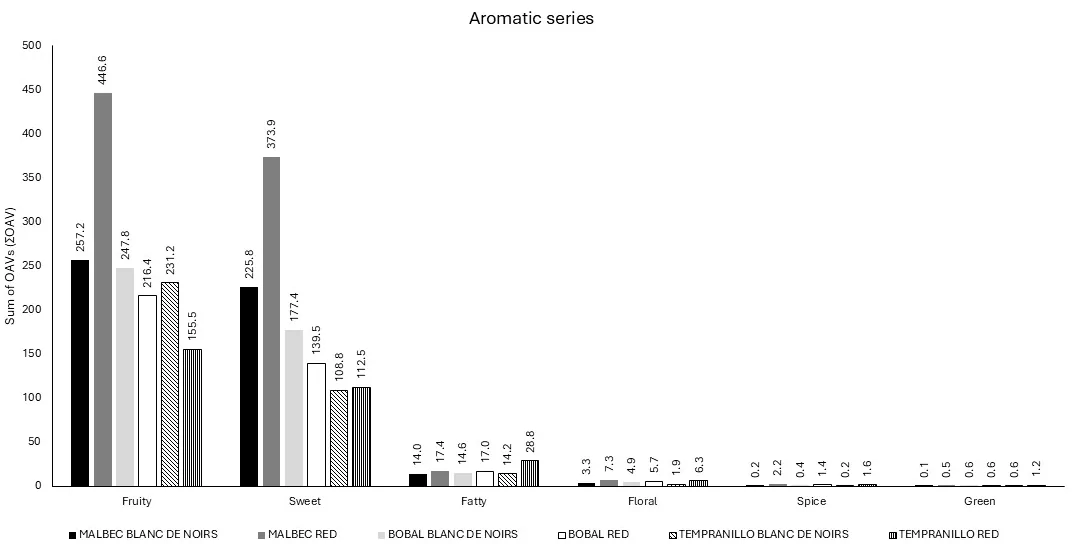
Aroma series calculated as the sum of the odour activity values of volatile compounds assigned to each descriptor category in blanc de noirs and traditional red Malbec, Bobal, and Tempranillo wines.

The intensities of the aromatic series, calculated as the sum of the OAVs of the compounds assigned to each category, are presented in Figure 3. The fruity and sweet series clearly predominated in all wines, although their intensities depended strongly on grape variety and vinification method. Malbec showed the highest values for both series, particularly after red vinification, mainly because of the high OAVs of β-damascenone and ethyl octanoate, together with the contributions of ethyl hexanoate, ethyl butanoate, isoamyl acetate, and ethyl decanoate. Bobal presented relatively high fruity series value under both vinification conditions, although red vinification reduced this series slightly because the decrease in β-damascenone and isoamyl acetate was not fully compensated by the higher OAVs of ethyl octanoate and ethyl hexanoate. Blanc de noirs Tempranillo wines also displayed a high fruity-series value, mainly associated with ethyl octanoate, ethyl hexanoate, ethyl butanoate, and isoamyl acetate, whereas red vinification reduced this series despite the appearance of an odour-active contribution from β-damascenone. The sweet series followed a similar pattern because several compounds, particularly β-damascenone, ethyl octanoate, ethyl decanoate, guaiacol, eugenol, and vanillin-related compounds, were assigned to this category; consequently, red Malbec showed the highest sweet series value, while blanc de noirs Tempranillo wines presented the lowest. The fatty series increased under the traditional red vinification protocol in Bobal and Tempranillo, although its relative contribution was lower than initially estimated after correction of the branched-chain acid OAVs. The floral series showed much lower summed OAVs and was primarily associated with 2-phenylethanol, geraniol, linalool, 2-phenylethyl acetate, β-citronellol, and, to a limited extent, 1-hexanol; red Malbec showed the highest floral value, whereas blanc de noirs Tempranillo wines presented the lowest. The spicy series was quantitatively minor in all samples because most of its constituent compounds, including eugenol, 4-vinylguaiacol, vanillin, ethyl vanillate, methyl vanillate, and acetovanillone, had individual OAVs below 1, although red vinification increased this series through the greater contribution of guaiacol and other volatile phenols. These summed OAVs provide an approximate indication of the potential sensory orientation of each wine, but they should not be regarded as direct measurements of perceived intensity because compounds assigned to the same descriptor may act additively or synergistically, whereas odorants with different or opposing descriptors may suppress or mask one another. Thus, the high fruity and sweet series values of red Malbec were consistent with a greater calculated contribution of compounds assigned to these aroma series. In contrast, red Bobal and red Tempranillo showed higher fatty series values, reflecting the greater calculated contribution of volatile acids to their OAV-based profiles. β-Damascenone may also enhance the perception of fruity odorants even when its isolated descriptor is not directly recognised, while fatty acids and smoky or phenolic compounds may compete with ester-derived notes. Wine aroma therefore emerges from cooperative, competitive, destructive, and creative interactions rather than from the arithmetic addition of individual OAVs, meaning that a high summed OAV does not necessarily correspond to an equally intense sensory attribute [18,23,51]. Consequently, the aromatic-series values in Figure 3 should be interpreted only as comparative indices of potential aroma contribution. Differences between the calculated aromatic series and actual sensory perception may arise from matrix effects, subthreshold interactions, suppression, or synergistic enhancement among odorants.

## 4. Conclusions

Blanc de noirs and traditional red vinification produced significantly different volatile profiles in wines made from Malbec, Bobal, and Tempranillo grapes, with significant grape variety × vinification interactions demonstrating cultivar-dependent responses. Red vinification generally increased the concentrations of grape-derived volatile compounds, higher alcohols, and volatile acids, whereas its effect on ester concentrations varied among cultivars. Malbec showed high estimated aroma potential, particularly after red vinification, although blanc de noirs Malbec wines maintained a distinctive volatile profile. Bobal was characterised by a relevant contribution of C_6_ compounds and esters, whereas Tempranillo showed the largest differences in OAV-based aroma series between vinification methods. β-Damascenone, ethyl octanoate, ethyl hexanoate, ethyl butanoate, isoamyl acetate, 2-phenylethanol, and several volatile fatty acids showed the greatest potential aroma relevance based on their OAVs. Nevertheless, individual and summed OAVs provide only an estimate of potential aroma contribution and cannot establish actual sensory perception because they do not fully account for additive, synergistic, antagonistic, or matrix-dependent interactions among volatile compounds. Overall, blanc de noirs and traditional red vinification produced different volatile profiles, and the response to vinification depended on grape cultivar. The results are restricted to volatile composition and estimated aroma potential and do not establish the sensory quality, consumer acceptance, or commercial suitability of the resulting wines. Further physicochemical, sensory, technological, and economic studies are therefore required to determine whether blanc de noirs production may represent a suitable approach for wine diversification in Castilla-La Mancha.

## Figures and Tables

**Table 1:** Values are expressed as mean (standard deviation n = 2). Must parameters were evaluated by one-way analysis of variance, followed by the Student–Newman–Keuls test when significant differences were detected. For wines, the effects of grape variety (V), vinification method (W), and their interaction (V × W) were evaluated by two-way analysis of variance. Exact *p*-values are reported.

	Musts												One-Way ANOVA *p*-Value					
	Malbec				Bobal				Tempranillo									
Soluble Solids (°Brix)	20.15		(0.07)		20.95		(0.04)		20.46		(0.35)		0.051					
pH	3.26 ^a^		(0.04)		3.88 ^b^		(0.01)		3.76 ^b^		(0.13)		0.008					
Titratable Acidity (g/L) *	5.43		(0.26)		5.31		(0.14)		5.48		(0.08)		0.663					
	**Wines**																	
	**Malbec**				**Bobal**				**Tempranillo**				**Two-way ANOVA** ***p*-value**			**partial eta squared (ηp^2^)**		
	**Blanc de Noirs**		**Red**		**Blanc de Noirs**		**Red**		**Blanc de Noirs**		**Red**		**V**	**W**	**V × W**	**V**	**W**	**V × W**
pH	3.55	(0.01)	3.77	(0.01)	3.41	(0.03)	3.27	0.217	<0.001	(0.12)	3.72	(0.06)	<0.001	0.217	<0.001	0.983	0.241	0.936
Ethanol (%*v*/*v*)	11.45	(0.07)	11.95	(0.07)	11.50	(0.28)	11.95	<0.001	0.367	(0.07)	11.95	(0.07)	0.367	<0.001	0.367	0.284	0.950	0.284
Titratable Acidity (g/L) *	4.73	(0.32)	4.95	(0.07)	4.78	(0.04)	5.05	0.013	0.785	(0.37)	4.80	(0.28)	0.036	0.013	0.785	0.671	0.666	0.077
Volatile Acidity (g/L) **	0.21	(0.01)	0.22	(0.01)	0.26	(0.06)	0.24	0.849	0.290	(0.05)	0.27	(0.06)	0.010	0.849	0.290	0.785	0.007	0.338
Free SO_2_ (mg/L)	15.50	(0.42)	15.40	(0.14)	15.70	(0.14)	15.60	0.067	0.234	(0.49)	16.70	(0.57)	<0.001	0.067	0.234	0.930	0.454	0.384
Total SO_2_ (mg/L)	40.75	(0.64)	43.15	(2.76)	41.25	(0.07)	43.65	0.003	0.272	(0.78)	42.65	(2.05)	0.607	0.003	0.272	0.153	0.797	0.352

* as tartaric acid; ** as acetic acid; ^a,b^ different superscript letters within the same row indicate significant differences among cultivars according to SNK test (*p* < 0.05).

**Table 2:** Odour descriptors, odour thresholds, aroma-series assignments, and odour activity values of volatile compounds in blanc de noirs and traditional red Malbec, Bobal, and Tempranillo wines.

COMPOUNDS	Odour Descriptors	Odour Threshold (μg L^−1^)	Aroma Series	MALBEC		BOBAL		TEMPRANILLO	
				BLANC DE NOIRS	RED	BLANC DE NOIRS	RED	BLANC DE NOIRS	RED
β-Damascenone	apple, floral, sweet	0.05	1, 4	111.92	251.22	92.91	22.50	0.00	69.10
Ethyl octanoate	caramel, fruity	5	1, 4	109.54	114.46	79.17	105.46	104.12	31.70
Ethyl butanoate	fruity	20	1	16.92	19.81	17.42	20.31	48.43	18.36
Isoamyl acetate	banana	30	1	8.75	19.03	50.20	35.79	11.45	11.39
Octanoic acid	sweat, cheese	500	6	8.51	9.54	7.87	6.30	7.46	8.27
Ethyl hexanoate	green apple	14	1	8.34	40.33	6.43	31.05	66.02	24.65
Hexanoic acid	sweat	1000	6	2.26	2.95	2.83	2.83	2.53	2.58
Isovaleric acid	acid, rancid	33	4, 6	1.66	4.79	2.75	5.22	2.78	7.33
Decanoic acid	rancid fat	1000	6	1.25	2.51	0.83	1.98	1.03	3.23
2-Phenylethanol	floral, roses, perfume	10.000	2	1.23	4.02	3.68	3.53	0.76	5.86
Ethyl decanoate	caramel, fruity	200	1, 4	1.78	1.77	1.64	1.30	1.16	0.29
Geraniol	rose, geranium	30	2	1.01	1.18	0.22	0.60	0.00	0.00
Guaiacol	medicine, caramel, smoke	10	4, 6	0.92	3.60	0.91	4.23	0.74	3.52
2-Phenylethyl acetate	floral	250	2	0.70	0.72	0.68	0.68	0.51	0.17
Linalool	floral	15	2	0.34	1.12	0.18	0.57	0.55	0.00
Butyric acid	rancid, cheese, sweat	173	6	0.21	0.05	0.25	0.11	0.35	0.65
Vanillin	vanillin	60	5, 7	0.21	0.36	0.26	0.07	0.13	0.08
3-Methylthio-1-propanol	cabbage, cooked potato	1000	6	0.04	0.41	0.02	0.51	0.06	0.46
(*Z*)-3-Hexen-1-ol	green, cut grass	400	3	0.03	0.09	0.43	0.32	0.38	0.83
Benzoic acid	chemical	1000	6	0.03	0.14	0.03	0.06	0.00	0.07
1-Hexanol	resin, flower, green, cut grass	8000	2, 3	0.02	0.22	0.05	0.15	0.08	0.18
(E)-3-Hexen-1-ol	green, cut grass	400	3	0.02	0.16	0.07	0.18	0.11	0.20
Ethyl vanillate	caramel, honey, vanillin	990	4, 5, 7	0.01	0.14	0.06	0.10	0.00	0.04
Acetovanillone	sweet spices	1000	5	0.01	0.45	0.03	0.06	0.02	0.04
β-Citronellol	Floral	40	2	0.00	0.00	0.08	0.19	0.00	0.08
Eugenol	spices, clove, honey	6	4, 5, 7	0.00	0.95	0.00	0.70	0.00	0.57
4-Vinylguaiacol	spicy/curry	40	5	0.00	0.31	0.00	0.51	0.00	0.91

OAVs were calculated as the ratio between the concentration of each compound and its corresponding odour threshold reported in the literature. Only compounds with an OAV > 0.1 in at least one wine were included. Aroma series: 1, fruity; 2, floral; 3, green/fresh; 4, spicy; 5, sweet; 6, fatty; and 7, other. Compounds assigned exclusively to the “other” category were not included in Figure 3.

## Data Availability

Data will be made available upon request.

## Supplementary Materials

The following supporting information can be downloaded at: https://www.mdpi.com/article/10.3390/foods15213999/s1, Table S1: Calibration and identification parameters for varietal volatile compounds; Table S2: Calibration and identification parameters for fermentation-derived volatile compounds; Table S3: Concentrations of varietal volatile compounds and two-way ANOVA results; Table S4: Concentrations of fermentation-derived volatile compounds and two-way ANOVA results. Tables S3 and S4 report exact *p*-values and partial eta squared values (ηp^2^) for grape variety, vinification method, and their interaction.
