# Supplementary material for: Effects of Blanc de Noirs and Traditional Red Vinification on the Volatile Composition and Estimated Aroma Potential of Malbec, Bobal, and Tempranillo Wines
Source: Foods. 2026 Nov 7;15(21):3999. doi: 10.3390/foods15213999 (PMC13762885; doi:10.3390/foods15213999)
Supplement: Supplementary file 1 [file foods-15-03999-s001.zip › foods-4627025-supplementary.pdf]

Table S1. Calibration parameters and identification information of varietal volatile compounds

Compounds	Calibration curves	R^2	Retention index (RI, calculated)	Linear range (mg/L)	LOD (mg/L)	LOQ (mg/L)	Source	Identification method
1-Hexanol	y=0.550x	0.9999	1282	0.050~2	0.002	0.005	Fluka	Authentic standard + RI + NBS75K+Wiley
(E)-3-hexen-1-ol	y=0.460x	0.9984	1286	0.005~0.500	0.001	0.004	Sigma-Aldrich	Authentic standard + RI + NBS75K+Wiley
(Z)-3-hexen-1-ol	y=0.410x	0.9991	1296	0.005~0.500	0.002	0.004	Sigma-Aldrich	Authentic standard + RI + NBS75K+Wiley
2-ethyl-1-hexanol	y=0.490x	0.9924	1394	0.002~0.400	0.002	0.003	Sigma-Aldrich	Authentic standard + RI + NBS75K+Wiley
(Z)-2-hexen-1-ol	y=0.420x	0.9915	1416	0.001~0.015	0.0006	0.001	Sigma-Aldrich	Authentic standard + RI + NBS75K+Wiley
cis-linalool- furan oxide			1455				Tentatively identified	RI + NBS75K+Wiley
Linalool	y=0.740x	0.9997	1529	0.002~0.025	0.001	0.002	Fluka	Authentic standard + RI + NBS75K+Wiley
α-terpineol	y=0.870x-0.07	0.9941	1715	0.002~0.043	0.001	0.002	Fluka	Authentic standard + RI + NBS75K+Wiley
β-citronellol	y=0.740x+0.002	0.9983	1755	0.002~0.015	0.001	0.0015	Fluka	Authentic standard + RI + NBS75K+Wiley
Nerol	y=1.030x	0.9981	1819	0.001~0.045	0.0005	0.001	Fluka	Authentic standard + RI + NBS75K+Wiley
Geraniol	y=0.980x-0.001	0.9931	1831	0.004~0.050	0.002	0.003	Fluka	Authentic standard + RI + NBS75K+Wiley
β-damascenone	y=0.921x	0.9941	1801	0.0005~0.020	0.00004	0.0004	Firmenich	Authentic standard + RI + NBS75K+Wiley
3-hydroxy β-damascone			2558				Tentatively identified	RI + NBS75K+Wiley
3-oxo-α-ionol			2582				Tentatively identified	RI + NBS75K+Wiley
4-oxo-7,8-dihydro- β-ionol			2672				Tentatively identified	RI + NBS75K+Wiley
7,8-dihydro-3-oxo- α-ionol			2875				Tentatively identified	RI + NBS75K+Wiley
Benzaldehyde	y=0.890x	0.9961	1503	0.002~0.020	0.001	0.002	Sigma-Aldrich	Authentic standard + RI + NBS75K+Wiley
Guaiacol	y=0.910x-0.004	0.9978	1882	0.005~0.100	0.002	0.004	Sigma-Aldrich	Authentic standard + RI + NBS75K+Wiley
Benzyl alcohol	y=1.090x	0.9971	1895	0.010~0.300	0.008	0.009	Sigma-Aldrich	Authentic standard + RI + NBS75K+Wiley
Phenol	y=1.001x-0.004	0.9996	1971	0.002~0.040	0.001	0.001	Sigma-Aldrich	Authentic standard + RI + NBS75K+Wiley
4-methoxyacetophenone	y=0.945x	0.9879	2148	0.010~0.250	0.005	0.009	Sigma-Aldrich	Authentic standard + RI + NBS75K+Wiley
Eugenol	y=0.579x-0.001	0.9934	2193	0.001~0.020	0.0005	0.0008	Sigma-Aldrich	Authentic standard + RI + NBS75K+Wiley

4-vinylguaiacol	y=0.840x+0.002	0.9986	2219	0.008~0.200	0.003	0.005	Sigma-Aldrich	Authentic standard + RI + NBS75K+Wiley
2,6-dimethoxy-phenol	y=0.759x-0.001	0.9974	2225	0.015~0.300	0.001	0.001	Sigma-Aldrich	Authentic standard + RI + NBS75K+Wiley
2,3-dihydrobenzofuran			2345				Tentatively identified	RI + NBS75K+Wiley
Benzoic acid	y=0.685x+0.012	0.9997	2378	0.010~0.200	0.005	0.009	Sigma-Aldrich	Authentic standard + RI + NBS75K+Wiley
Benzeneacetic acid			2501				Tentatively identified	RI + NBS75K+Wiley
Vanillin	y=0.743x	0.9997	2511	0.004~0.175	0.002	0.004	Panreac	Authentic standard + RI + NBS75K+Wiley
Methyl vanillate	y=0.885x+0.004	0.9997	2543	0.001~0.150	0.002	0.003	Sigma-Aldrich	Authentic standard + RI + NBS75K+Wiley
Ethyl vanillate	y=0.756x	0.9997	2676	0.005~0.300	0.0015	0.002	Lancaster	Authentic standard + RI + NBS75K+Wiley
Acetovanillone	y=0.718x-0.002	0.9997	2685	0.005~0.500	0.002	0.004	Sigma-Aldrich	Authentic standard + RI + NBS75K+Wiley
Zingerone	y=0.685x+0.012	0.9997	2936	0.001~0.150	0.001	0.003	Sigma-Aldrich	Authentic standard + RI + NBS75K+Wiley
Methyl vanillyl ether			3030				Tentatively identified	RI + NBS75K+Wiley

RI retention index determined on a DB-WAX capillary column

Table S2. Calibration parameters and identification information of volatile compounds formed principally during fermentation

Compounds	Calibration curves	R^2	Retention index (RI, calculated)	Linear range (mg/L)	LOD (mg/L)	LOQ (mg/L)	Source	Identification method
Ethyl butanoate	y=0.9812x	0.998	1080	0.005~12	0.002	0.005	Fluka	Authentic standard + RI + NBS75K+Wiley
Isoamyl acetate	y = 1.0532x	0.997	1145	0.006~24	0.001	0.006	Sigma-Aldrich	Authentic standard + RI + NBS75K+Wiley
Ethyl hexanoate	y=0.7986x	0.995	1185	0.004~15	0.001	0.003	Fluka	Authentic standard + RI + NBS75K+Wiley
Hexyl acetate	y=0.5834x	0.997	1294	0.002~0.500	0.001	0.002	Sigma-Aldrich	Authentic standard + RI + NBS75K+Wiley
Ethyl lactate	y=0.0432x	0.99	1326	0.002~0.600	0.002	0.002	Sigma-Aldrich	Authentic standard + RI + NBS75K+Wiley
Ethyl octanoate	y=0.4517x	0.996	1436	0.002~0.600	0.001	0.002	Sigma-Aldrich	Authentic standard + RI + NBS75K+Wiley
Ethyl decanoate	y = 0.9995x	1	1655	0.02~10	0.001	0.003	Fluka	Authentic standard + RI + NBS75K+Wiley
Diethyl succinate	y=0.7356x+0.0123	1	1702	0.004~10	0.002	0.006	Fluka	Authentic standard + RI + NBS75K+Wiley
Ethyl 4-hydroxybutyrate			1827				Tentatively identified	RI + NBS75K+Wiley
2-phenylethyl acetate	y=0.837x-0.2132	0.998	1825	0.03~5	0.001	0.004	Sigma-Aldrich	Authentic standard + RI + NBS75K+Wiley
Diethyl malate	y=0.9343x	1	2070	0.010~0.200	0.005	0.008	Sigma-Aldrich	Authentic standard + RI + NBS75K+Wiley
Ethyl monosuccinate			2331				Tentatively identified	RI + NBS75K+Wiley
Acetic acid	y=0.09160x	0.9996	1426	0.002~0.150	0.001	0.002	Sigma-Aldrich	Authentic standard + RI + NBS75K+Wiley
Isobutyric acid	y=0.0871x	0.9879	1583	0.010~2	0.005	0.007	Fluka	Authentic standard + RI + NBS75K+Wiley
Butyric acid	y=0.048x	0.993	1600	0.002~2.5	0.001	0.005	Fluka	Authentic standard + RI + NBS75K+Wiley
Isovaleric acid	y=0.057x-0.123	0.999	1642	0.03~3	0.020	0.025	Sigma-Aldrich	Authentic standard + RI + NBS75K+Wiley
2-methylbutanoic acid	y = 0.232x - 0.221	1	1662	0.025~3	0.010	0.020	Sigma-Aldrich	Authentic standard + RI + NBS75K+Wiley
Pentanoic acid	y = 0.6431x	0.998	1703	0.002~0.050	0.001	0.002	Fluka	Authentic standard + RI + NBS75K+Wiley

Hexanoic acid	y = 0.5287x - 0.012	0.997	1816	2~10	0.005	0.015	Fluka	Authentic standard + RI + NBS75K+Wiley
(E)-2-hexenoic acid	y = 0.634x	0.997	1929	0.002~0.200	0.001	0.002	Sigma-Aldrich	Authentic standard + RI + NBS75K+Wiley
(E)-3-hexenoic acid	y = 0.539x -0.23	0.995	1957	0.010~0.200	0.002	0.005	Sigma-Aldrich	Authentic standard + RI + NBS75K+Wiley
Octanoic acid	y =0.6321x	0.996	2024	0.001~5	0.001	0.004	Fluka	Authentic standard + RI + NBS75K+Wiley
Decanoic acid	y = 0.7625x	0.992	2289	0.5~10	0.001	0.004	Sigma-Aldrich	Authentic standard + RI + NBS75K+Wiley
Dodecanoic acid	y = 0.8245x	0.999	2439	0.030~0.200	0.005	0.01	Sigma-Aldrich	Authentic standard + RI + NBS75K+Wiley
1-Butanol	y = 0.297x + 0.0134	0.998	1155	0.008~0.350	0.010	0.030	Sigma-Aldrich	Authentic standard + RI + NBS75K+Wiley
2-methyl-1-butanol	y = 0.356x + 0.0241	0.997	1221	0.025~1	0.01	0.02	Sigma-Aldrich	Authentic standard + RI + NBS75K+Wiley
3-methyl-1-butanol	y = 0.298x +0.0148	0.998	1221	0.001~1	0.001	0.001	Sigma-Aldrich	Authentic standard + RI + NBS75K+Wiley
1-Pentanol	y = 0.345x	0.992	1260	0.009~0.200	0.004	0.005	Sigma-Aldrich	Authentic standard + RI + NBS75K+Wiley
3-methyl-1-pentanol	y = 0.298x +0.0148	0.995	1328	0.010~0.300	0.005	0.008	Fluka	Authentic standard + RI + NBS75K+Wiley
2,3-Butanediol (*levo*)	y = 0.587x-0.045	0.99	1545	0.006~0.300	0.004	0.005	Fluka	Authentic standard + RI + NBS75K+Wiley
2,3-Butanediol (*meso*)	y=0.674x-0.067	0.992	1585	0.006~0.300	0.004	0.004	Fluka	Authentic standard + RI + NBS75K+Wiley
3-Methylthio-1-propanol	y = 0.125x-0.051	0.999	1725	0.01~1	0.005	0.007	Sigma-Aldrich	Authentic standard + RI + NBS75K+Wiley
2-Phenylethanol	y = 0.441x	0.996	1892	5~80	1	1.5	Fluka	Authentic standard + RI + NBS75K+Wiley

RI retention index determined on a DB-WAX capillary column

Table S3. Mean concentrations (µg L^{-1}) and relative standard deviation (n=2) of varietal volatile compounds in Malbec, Bobal, and Tempranillo wines produced by blanc de noirs and traditional red vinification, and results of the two-way analysis of variance evaluating the effects of grape variety, vinification method, and their interaction.

Compounds	MALBEC				BOBAL				TEMPRANILLO				Two-way ANOVA p-value			Partial eta squared (ηp²)		
	BLANC DE NOIRS		RED		BLANC DE NOIRS		RED		BLANC DE NOIRS		RED		V	W	V × W	V	W	V × W
1-Hexanol	166.26	(0.86)	1754.56	(7.23)	434.82	(0.05)	1180.24	(0.66)	664.64	(6.90)	1460.55	(2.67)	< 0.001	< 0.001	< 0.001	0.989	0.999	0.991
(E)-3-hexen-1-ol	8.56	(9.48)	63.66	(6.30)	27.71	(1.68)	71.45	(0.73)	42.69	(1.17)	79.02	(0.04)	< 0.001	< 0.001	< 0.001	0.999	1.000	0.983
(Z)-3-hexen-1-ol	10.12	(2.94)	34.12	(7.80)	173.40	(4.54)	129.07	(1.69)	153.97	(9.12)	332.86	(0.10)	< 0.001	0.024	0.005	0.992	0.341	0.882
2-ethyl-1-hexanol	6.35	(6.33)	9.02	(4.59)	3.97	(0.24)	9.07	(1.09)	17.16	(9.26)	11.98	(4.96)	< 0.001	0.001	0.175	0.986	0.906	0.404
(Z)-2-hexen-1-ol	1.91	(7.84)	12.86	(7.29)	3.47	(1.19)	n.d.	-	7.28	(8.95)	n.d.	-	< 0.001	< 0.001	< 0.001	0.994	0.976	0.994
Total C_6 compounds	193.20		1874.23		643.38		1389.82		885.74		1884.41							
cis-linalool furan oxide	3.57	(2.51)	9.16	(6.10)	1.82	(0.54)	n.d.	-	3.84	(1.32)	2.80	(3.79)	< 0.001	< 0.001	< 0.001	1.000	0.998	1.000
Linalool	5.10	(0.52)	16.73	(1.71)	2.69	(0.63)	8.57	(1.65)	8.32	(4.00)	n.d.	-	< 0.001	< 0.001	< 0.001	0.998	0.999	0.991
α-terpineol	5.73	(6.73)	22.31	(0.85)	14.32	(6.15)	n.d.	-	n.d.	-	10.44	(2.30)	< 0.001	0.035	< 0.001	0.996	0.712	0.998
β-citronellol	n.d.	-	n.d.	-	3.07	(0.94)	7.66	(1.57)	n.d.	-	3.39	(3.34)	< 0.001	< 0.001	0.014	0.993	0.995	0.816
Nerol	4.74	(9.49)	10.70	(2.43)	5.49	(1.89)	1.28	(2.21)	n.d.	-	4.43	(3.83)	< 0.001	< 0.001	< 0.001	1.000	0.998	0.998
Geraniol	30.42	(2.51)	35.44	(7.52)	6.71	(1.12)	17.89	(1.70)	n.d.	-	n.d.	-	< 0.001	< 0.001	< 0.001	0.999	0.992	0.974
Total terpene	49.56		94.34		34.10		35.39		12.16		21.06							
beta-damascenone	5.60	(6.20)	12.56	(1.45)	4.65	(1.67)	1.13	(3.14)	n.d.	-	3.46	(5.94)	< 0.001	< 0.001	< 0.001	0.999	0.972	0.997
3-hydroxy-*beta* damascone	n.d.	-	n.d.	-	n.d.	-	1.21	(2.93)	n.d.	-	3.96	(3.75)	< 0.001	< 0.001	< 0.001	0.998	0.998	0.998
3-oxo-*alpha*-ionol	n.d.	-	63.93	(4.39)	47.88	(1.71)	29.53	(1.70)	27.23	(7.72)	15.10	(3.65)	< 0.001	< 0.001	< 0.001	0.954	0.993	0.998
4-oxo-7,8-dihydro-β-ionol	n.d.	-	12.83	(2.43)	n.d.	-	n.d.	-	n.d.	-	n.d.	-	< 0.001	< 0.001	< 0.001	1.000	1.000	1.000
7,8-dihydro-3-oxo-α-ionol	n.d.	-	118.76	(2.43)	n.d.	-	n.d.	-	n.d.	-	n.d.	-	< 0.001	< 0.001	< 0.001	1.000	1.000	1.000
Total C_{13}-norisoprenoids	5.60		208.08		52.52		31.86		27.23		22.51							
Benzaldehyde	n.d.	-	n.d.	-	7.67	(1.37)	4.70	(0.30)	2.12	(8.43)	4.99	(0.57)	< 0.001	< 0.001	< 0.001	0.999	0.988	0.988

Guaiacol	9.17	(2.55)	36.02	(4.31)	9.11	(8.12)	42.31	(2.12)	7.40	(7.13)	35.20	(1.89)	< 0.001	0.197	0.004	0.996	0.374	0.900
Benzyl alcohol	18.07	(4.19)	211.89	(6.42)	80.92	(1.46)	151.71	(11.16)	41.46	(6.80)	228.89	(0.65)	< 0.001	< 0.001	< 0.001	0.999	1.000	0.999
Phenol	5.54	(1.29)	8.03	(2.43)	14.63	(1.43)	19.08	(1.96)	10.20	(6.56)	21.03	(1.04)	< 0.001	< 0.001	0.045	0.996	0.969	0.675
4-methoxyacetophenone	18.49	(4.12)	221.10	(0.82)	177.12	(6.83)	40.24	(2.41)	100.01	(4.08)	n.d.	-	0.225	0.003	< 0.001	0.525	0.910	0.996
Eugenol	n.d.	-	5.69	(12.06)	n.d.	-	4.19	(0.51)	n.d.	-	3.41	(0.83)	< 0.001	< 0.001	< 0.001	1.000	1.000	1.000
4-vinylguaiacol	n.d.	-	12.51	(4.86)	n.d.	-	20.51	(2.96)	n.d.	-	36.51	(3.64)	< 0.001	< 0.001	< 0.001	0.998	0.998	0.998
2,6-dimethoxyphenol	n.d.	-	154.53	(2.50)	152.00	(1.26)	177.85	(3.91)	109.30	(0.39)	249.00	(0.53)	< 0.001	< 0.001	< 0.001	0.996	0.996	0.992
2,3-dihydrobenzofuran	62.58	(2.36)	397.99	(1.86)	158.03	(1.09)	358.03	(0.48)	n.d.	-	n.d.	-	< 0.001	< 0.001	< 0.001	1.000	1.000	1.000
Benzoic acid	27.90	(0.71)	138.38	(5.98)	25.81	(2.03)	62.99	(1.12)	n.d.	-	70.27	(1.98)	< 0.001	< 0.001	< 0.001	1.000	1.000	1.000
Benzeneacetic acid	25.46	(5.96)	98.11	(2.28)	13.63	(1.76)	34.13	(0.25)	16.41	(4.28)	67.48	(2.64)	< 0.001	< 0.001	< 0.001	0.999	0.999	0.998
Vanillin	12.39	(4.28)	21.86	(2.81)	15.66	(1.13)	4.15	(7.34)	7.92	(0.38)	4.92	(2.45)	< 0.001	0.033	< 0.001	0.997	0.717	0.998
Methyl vanillate	n.d.	-	n.d.	-	14.32	(1.53)	2.20	(4.50)	n.d.	-	2.39	(0.30)	< 0.001	< 0.001	< 0.001	1.000	0.999	0.999
Ethyl vanillate	8.13	(2.51)	140.60	(0.09)	64.11	(2.06)	100.02	(3.68)	n.d.	-	37.91	(0.45)	< 0.001	< 0.001	< 0.001	0.992	0.999	0.996
Acetovanillone	10.54	(2.51)	445.81	(2.43)	34.47	(1.17)	58.81	(5.12)	24.98	(9.16)	36.69	(3.01)	< 0.001	< 0.001	< 0.001	1.000	1.000	1.000
Zingerone	n.d.	-	n.d.	-	16.77	(4.80)	4.88	(1.74)	11.76	(4.52)	2.40	(1.18)	< 0.001	< 0.001	< 0.001	0.995	0.984	0.984
Methyl vanillil ether	n.d.	-	n.d.	-	n.d.	-	26.71	(0.82)	n.d.	-	18.67	(4.66)	< 0.001	< 0.001	< 0.001	1.000	1.000	1.000
Total benzenic compounds	198.29		1892.50		784.25		1112.48		331.57		819.74							

V, grape variety; W, vinification method; V × W, interaction between grape variety and vinification method; n.d., not detected. Exact p-values and partial eta squared values (ηp^2) are reported for each effect.

Table S4. Mean concentrations (µg L^{-1}) and relative standard deviation (n=2) of volatile compounds formed principally during alcoholic fermentation in Malbec, Bobal, and Tempranillo wines produced by blanc de noirs and traditional red vinification, and results of the two-way analysis of variance evaluating the effects of grape variety, vinification method, and their interaction.

COMPOUNDS	MALBEC				BOBAL				TEMPRANILLO				Two-way ANOVA p-value			Partial eta squared (ηp²)		
	BLANC DE NOIRS		RED		BLANC DE NOIRS		RED		BLANC DE NOIRS		RED		V	W	V × W	V	W	V × W
Acetic acid	9.82	(3.95)	7.35	(2.73)	4.24	(0.03)	25.77	(1.59)	4.52	(2.27)	18.37	(0.58)	< 0.001	< 0.001	< 0.001	0.996	0.998	0.999
Isobutyric acid	14.34	(3.96)	12.07	(3.46)	11.74	(8.18)	20.62	(1.69)	24.47	(5.18)	164.13	(0.84)	< 0.001	< 0.001	< 0.001	0.999	0.999	0.999
Butyric acid	37.07	(3.81)	8.79	(2.15)	42.83	(0.00)	19.29	(1.69)	59.94	(7.55)	113.07	(1.75)	< 0.001	< 0.001	< 0.001	0.999	0.999	0.999
Isovaleric acid	54.79	(6.42)	158.09	(2.46)	90.82	(2.36)	172.33	(1.69)	91.88	(8.98)	241.82	(3.50)	< 0.001	< 0.001	< 0.001	0.999	0.999	0.999
2-methylbutanoic acid	83.06	(1.36)	181.21	(2.15)	39.99	(1.44)	196.33	(2.65)	61.95	(8.15)	201.32	(4.65)	< 0.001	< 0.001	< 0.001	0.999	0.982	0.997
Pentanoic acid	6.59	(3.35)	13.84	(2.43)	3.64	(0.54)	21.92	(2.68)	5.75	(5.31)	20.35	(1.32)	0.003	0.001	< 0.001	0.954	0.998	0.990
Hexanoic acid	2256.15	(4.60)	2950.99	(3.29)	2832.78	(0.76)	2827.81	(0.90)	2531.42	(4.18)	2576.39	(0.14)	< 0.001	< 0.001	< 0.001	0.990	0.966	0.966
(E)-2-hexenoic acid	5.86	(2.51)	124.74	(0.14)	26.92	(1.82)	144.02	(0.70)	22.02	(3.54)	135.06	(1.26)	< 0.001	< 0.001	< 0.001	1.000	1.000	1.000
(E)-3-hexenoic acid	10.59	(2.51)	11.69	(2.43)	14.59	(5.97)	15.64	(2.35)	17.71	(4.52)	14.12	(3.95)	< 0.001	< 0.001	< 0.001	0.976	0.980	0.986
Octanoic acid	4252.87	(1.31)	4769.96	(1.59)	3936.34	(0.27)	3152.16	(3.72)	3730.16	(8.42)	4134.86	(4.20)	< 0.001	< 0.001	< 0.001	0.996	0.995	0.987
Decanoic acid	1253.43	(2.51)	2506.26	(7.95)	825.97	(0.69)	1980.66	(1.99)	1027.61	(1.16)	3234.86	(2.31)	< 0.001	< 0.001	< 0.001	0.999	0.999	0.658
Dodecanoic acid	73.55	(7.42)	78.55	(15.95)	44.16	(4.53)	82.66	(0.28)	112.50	(3.22)	107.50	(3.21)	< 0.001	0.002	< 0.001	0.987	0.928	0.995
Total acids	8058.12		10823.54		7874.01		8659.19		7689.92		10961.82							
1-butanol	11.51	(14.84)	35.69	(14.15)	11.01	(9.10)	30.69	(6.59)	8.48	(6.68)	22.35	(0.89)	< 0.001	< 0.001	0.039	0.992	0.966	0.966
2-Methyl-1-butanol	105.13	(2.51)	577.42	(0.85)	38.01	(1.35)	534.01	(4.76)	103.96	(8.62)	582.93	(4.18)	< 0.001	< 0.001	< 0.001	0.989	0.992	0.993
3-methyl-1-butanol	19.82	(4.74)	17.07	(7.79)	4.29	(1.54)	13.51	(8.28)	3.42	(9.30)	20.83	(4.82)	< 0.001	< 0.001	< 0.001	0.998	0.963	0.127
1-Pentanol	12.01	(8.34)	37.19	(7.87)	13.51	(1.81)	19.96	(4.82)	10.53	(5.06)	15.82	(3.62)	< 0.001	< 0.001	0.002	0.997	0.929	0.929
3-methyl-1-pentanol	18.42	(2.51)	14.77	(2.43)	52.10	(0.55)	89.99	(1.10)	81.32	(7.40)	178.95	(4.94)	< 0.001	< 0.001	0.001	1.000	0.927	0.948
2,3-butanediol (levo)	6.58	(2.96)	98.13	(3.04)	6.51	(4.53)	13.04	(1.79)	6.58	(2.51)	7.95	(0.98)	< 0.001	< 0.001	< 0.001	0.999	0.999	0.999
2,3-butanediol (meso)	6.76	(2.51)	91.18	(3.07)	6.16	(3.25)	86.62	(1.85)	7.51	(3.92)	60.97	(3.64)	< 0.001	< 0.001	0.005	0.986	1.000	0.884

3-methylthio-1-propanol	44.73	(0.11)	406.12	(0.87)	19.66	(6.46)	513.83	(2.12)	60.81	(0.06)	463.83	(12.89)	< 0.001	< 0.001	< 0.001	0.981	0.982	0.982
2-Phenylethanol	12324.45	(2.08)	40236.90	(6.85)	36836.32	(0.03)	35320.33	(0.80)	7556.15	(8.80)	58633.75	(6.20)	< 0.001	< 0.001	0.014	0.983	1.000	0.812
Total alcohols	12549.42		41514.47		36987.57		36621.97		7838.76		59987.37							
Ethyl butanoate	338.38	(2.95)	396.23	(0.60)	348.38	(1.19)	406.23	(1.15)	968.66	(4.44)	367.11	(0.63)	< 0.001	< 0.001	0.003	0.990	0.953	0.953
Isoamyl acetate	262.36	(2.80)	570.97	(3.41)	1505.96	(1.30)	1073.79	(0.69)	343.49	(6.79)	341.81	(3.19)	< 0.001	< 0.001	< 0.001	0.999	0.879	0.995
Ethyl hexanoate	116.77	(1.36)	564.69	(4.09)	90.01	(1.62)	434.69	(4.45)	924.25	(9.70)	345.12	(2.02)	< 0.001	< 0.001	< 0.001	0.991	0.969	0.405
Hexyl acetate	17.39	(8.55)	8.97	(9.40)	35.58	(0.63)	5.13	(8.55)	7.45	(7.00)	5.83	(0.00)	< 0.001	< 0.001	< 0.001	0.995	0.995	0.987
Ethyl lactate	47.34	(6.86)	338.22	(2.43)	455.27	(1.64)	553.14	(5.02)	280.97	(6.91)	198.03	(2.47)	< 0.001	< 0.001	< 0.001	0.982	0.982	0.981
Ethyl octanoate	547.70	(8.30)	572.29	(2.05)	395.86	(0.25)	527.29	(1.64)	520.60	(3.59)	158.49	(0.00)	< 0.001	< 0.001	< 0.001	0.984	0.994	0.997
Ethyl decanoate	356.54	(0.48)	353.92	(0.14)	327.20	(0.05)	259.39	(0.41)	231.88	(2.92)	57.30	(0.99)	< 0.001	< 0.001	< 0.001	1.000	1.000	1.000
Diethyl succinate	236.24	(3.64)	637.70	(4.16)	279.78	(2.62)	654.61	(6.84)	437.88	(8.38)	210.81	(9.62)	< 0.001	< 0.001	< 0.001	0.995	0.996	0.522
Ethyl 4-hydroxybutyrate	227.50	(4.26)	407.58	(1.55)	93.25	(8.11)	341.19	(1.69)	166.40	(7.56)	55.91	(3.64)	< 0.001	< 0.001	< 0.001	0.999	0.999	0.999
2-phenylethyl acetate	174.23	(4.44)	178.90	(8.56)	169.73	(0.81)	168.90	(0.69)	127.99	(1.89)	41.44	(3.65)	< 0.001	< 0.001	< 0.001	1.000	0.998	0.998
Diethyl malate	63.74	(1.18)	33.38	(3.71)	105.03	(2.75)	139.69	(4.10)	129.05	(1.60)	64.92	(0.97)	< 0.001	< 0.001	< 0.001	0.993	0.951	0.944
Ethyl monosuccinate	350.50	(2.22)	2067.00	(3.63)	428.06	(9.93)	4426.86	(1.69)	601.15	(7.75)	910.97	(3.64)	< 0.001	< 0.001	< 0.001	0.999	0.999	0.999
Total esters	2738.69		6129.83		4234.10		8990.88		4739.77		2757.75							

V, grape variety; W, vinification method; V × W, interaction between grape variety and vinification method; n.d., not detected. Exact p-values and partial eta squared values (ηp^2) are reported for each effect.
